# Phosphorylation of the amyloid β-peptide at Ser26 stabilizes oligomeric assembly and increases neurotoxicity

**DOI:** 10.1007/s00401-016-1546-0

**Published:** 2016-02-22

**Authors:** Sathish Kumar, Oliver Wirths, Kathrin Stüber, Patrick Wunderlich, Philipp Koch, Sandra Theil, Nasrollah Rezaei-Ghaleh, Markus Zweckstetter, Thomas A. Bayer, Oliver Brüstle, Dietmar R. Thal, Jochen Walter

**Affiliations:** Department of Neurology, University of Bonn, 53127 Bonn, Germany; Division of Molecular Psychiatry, Department of Psychiatry and Psychotherapy, University Medical Center (UMG), Georg-August-University Göttingen, Von-Siebold-Str. 5, 37075 Göttingen, Germany; Institute of Reconstructive Neurobiology, University of Bonn, 53127 Bonn, Germany; LIFE & BRAIN Center, University of Bonn, 53127 Bonn, Germany; German Center for Neurodegenerative Diseases (DZNE), 37077 Göttingen, Germany; Department for NMR-Based Structural Biology, Max Planck Institute for Biophysical Chemistry, 37077 Göttingen, Germany; Center for the Molecular Physiology of the Brain, University Medical Center, 37077 Göttingen, Germany; German Center for Neurodegenerative Diseases (DZNE), 53175 Bonn, Germany; Laboratory of Neuropathology-Institute of Pathology, University of Ulm, 89081 Ulm, Germany

**Keywords:** Alzheimer’s disease, Phosphorylation, Protein aggregation, Intraneuronal Abeta, Amyloid oligomer, Granulovacuolar degeneration

## Abstract

Aggregation and toxicity of the amyloid β-peptide (Aβ) are considered as critical events in the initiation and progression of Alzheimer’s disease (AD). Recent evidence indicated that soluble oligomeric Aβ assemblies exert pronounced toxicity, rather than larger fibrillar aggregates that deposit in the forms of extracellular plaques. While some rare mutations in the Aβ sequence that cause early-onset AD promote the oligomerization, molecular mechanisms that induce the formation or stabilization of oligomers of the wild-type Aβ remain unclear. Here, we identified an Aβ variant phosphorylated at Ser26 residue (pSer26Aβ) in transgenic mouse models of AD and in human brain that shows contrasting spatio-temporal distribution as compared to non-phosphorylated Aβ (npAβ) or other modified Aβ species. pSer26Aβ is particularly abundant in intraneuronal deposits at very early stages of AD, but much less in extracellular plaques. pSer26Aβ assembles into a specific oligomeric form that does not proceed further into larger fibrillar aggregates, and accumulates in characteristic intracellular compartments of granulovacuolar degeneration together with TDP-43 and phosphorylated tau. Importantly, pSer26Aβ oligomers exert increased toxicity in human neurons as compared to other known Aβ species. Thus, pSer26Aβ could represent a critical species in the neurodegeneration during AD pathogenesis.

**Electronic supplementary material:**

The online version of this article (doi:10.1007/s00401-016-1546-0) contains supplementary material, which is available to authorized users.

## Introduction

Alzheimer’s disease (AD) is the most common form of dementia and characterized by the combined occurrence of extracellular amyloid plaques and intraneuronal neurofibrillary tangles [44]. The accumulation of amyloid-β (Aβ) as oligomers and fibrils is an early event in the development of AD. Aβ peptides derive from the proteolytic processing of the amyloid precursor protein (APP) by β- and γ-secretases [54]. A critical role of Aβ in the pathogenesis of AD is strongly supported by mutations in the genes encoding APP or presenilin 1 and 2 that cause early-onset familial forms of AD (FAD) [45]. These mutations commonly increase the production and/or aggregation of Aβ and deposition of amyloid plaques [7, 9, 18]. However, the vast majority of cases occur late in life without mutations in the amyloid precursor protein (APP) or presenilins (PS) that cause familial forms of early-onset AD.

The Aβ peptide is natively unfolded and tends to aggregate into soluble oligomers, protofibrils and fibrils [3]. Recent studies suggest that the toxicity of Aβ and other amyloidogenic proteins is not only exerted by insoluble fibrils, but rather by soluble oligomeric intermediates [11, 19, 33, 52]. Strong evidence indicates a critical role of soluble Aβ oligomers in the pathogenesis of AD [4, 19,32]. While extracellular deposits of this peptide in form of plaques only weakly correlate with neuronal cell death and clinical stage of AD, soluble oligomers [11, 32, 52] and intracellular [17, 56] deposits of Aβ have been shown to associate more closely with disease progression. Certain FAD mutations in the Aβ domain facilitate the formation of such assemblies [13, 23, 25, 34, 49]. However, these mutations are rare and mechanisms that drive the aggregation of wild-type Aβ during the pathogenesis of much more common sporadic forms of AD are largely unclear.

We recently demonstrated that extracellular Aβ undergoes phosphorylation by secreted variants of protein kinase A [26]. Phosphorylation of Aβ at serine (Ser) 8 residue promotes its aggregation into oligomeric and fibrillar assemblies [26]. Phosphorylation of Ser8 also attenuated the proteolytic degradation of Aβ by certain proteases and clearance by microglial cells [27]. By employing pSer8Aβ-specific monoclonal antibodies, we showed the early intraneuronal accumulation and increased aggregation of pSer8Aβ in transgenic mouse and human brains [29, 42]. These findings highlight the plausible role of Aβ phosphorylation in AD pathogenesis.

Aβ can also undergo phosphorylation at Ser26 which modulates its aggregation in vitro [36, 41]. Here we investigated the effect of Ser26 phosphorylation on aggregation, toxicity and its presence in human AD brains and transgenic mouse models. We demonstrate a peculiar deposition of Ser26 phosphorylated Aβ in human and transgenic mouse brain that differs from that observed for other Aβ species. Notably, phosphorylation of Aβ at Ser26 strongly promotes the formation and stabilization of low molecular weight soluble Aβ oligomers with increased toxicity on human neurons.

## Materials and methods

### Reagents and antibodies

Synthetic non-phosphorylated Aβ1–40 (npAβ), phosphorylated Aβ1–40 variants (pSer8Aβ and pSer26Aβ) and other modified Aβ (Tyr10 nitrated, Glu3 pyroglutamate and truncated 3–42) peptides were purchased from Peptide Speciality Laboratory (PSL, Germany). Thioflavin T, 4′,6-diamidino-2′phenylindole dihydrochloride (DAPI), 3,3′-diamino-benzidine (DAB) and methanol were from Sigma-Aldrich (USA). Congo red was purchased from AppliChem GmbH (Germany). Precast 4–12 % NuPAGE Bis–Tris mini and midi gels, prestained protein molecular weight markers and PrestoBlue^®^ cell viability reagent were from Life technologies (Germany). Nitrocellulose membranes were from Schleicher and Schuell (Germany). ECL Western blotting detection reagents were from GE Healthcare (UK). Vectastain ABC kit and hematoxylin were from Vector laboratories (USA). Protease and phosphatase inhibitors were from Roche laboratories (Germany). BCA™ protein assay kit was from Thermo Scientific (USA). Monoclonal Aβ antibodies 6E10 and 4G8 were purchased from Covance Laboratories (USA), and 82E1 antibody was from IBL Corporation (Japan). Mouse monoclonal GFAP antibody was from Synaptic systems (Germany), and 22C11 antibody specific against amyloid precursor protein (APP) (a.a. 66–81 of APP at N-terminus) was from Merck Millipore (Germany). A Mouse monoclonal Phospho-PHF-tau specific AT8 antibody was purchased from Thermo scientific (USA). Rabbit polyclonal anti-CK1δ (antiserum 108) and anti-CK1ε (antiserum 712) were generously provided by Dr. Uwe Knippschild from University Hospital Ulm, Germany. The anti-mouse, anti-rabbit secondary antibodies conjugated to horseradish peroxidase were from Sigma Aldrich (Germany), Secondary fluorescent anti-mouse 594 DyLight, anti-rabbit 488 antibodies were from Thermo Scientific (USA), IRDye800CW and IRDye680RD were from LI-COR Biotechnology. Biotinylated secondary anti-mouse and anti-rabbit antibodies were from DAKO (Glostrup, Denmark). The dilutions of each antibody stock are mentioned for the respective methods or in figure legends.

### Generation of pSer26Aβ-specific antibodies

The pSer26Aβ-specific polyclonal antibody SA6192 was generated in rabbits by injecting synthetic Aβ19–31 peptides with Ser26 in phosphorylated state (antigen sequence: FFAEDVG (p) SNKGAI) conjugated with keyhole limpet hemocyanin (KLH) (Eurogentec, Belgium). Phosphorylation state-specific antibodies were purified from the serum by double-affinity purification using pSer26Aβ and npAβ peptide. The specificity of the purified antibodies was characterized by enzyme-linked immunosorbent assay (ELISA) and Western blotting (WB). Further details are described in the Supplementary Information.

### Biochemical and immunohistochemical detection of pSer26Aβ in transgenic mouse brains

For biochemical analysis of pSer26Aβ, whole brain homogenates from APP/PS1KI were prepared as described previously [26, 29]. Immunohistochemistry was performed on 4 µm sagittal paraffin sections as described previously [58]. Further details of Aβ extraction and immunohistochemistry of transgenic mouse brains are described in the Supplementary Information.

### Immunohistochemistry of human AD brain

Human autopsy brains were received from University Hospital Bonn (Germany) and from University Hospital Ulm (Germany) in accordance with the laws and the permission of the local ethical committees. Post-mortem diagnosis of Alzheimer’s disease was carried out according to the NIA-Reagan Criteria [6, 37]. All procedures were conducted in accordance with the laws and the permission of the local ethical committees. Further detailed methods and information on cases are given in the Supplementary Information.

### Aβ aggregation assays

Aβ aggregation kinetics by Thioflavin T (ThT) and Congo Red (CR) binding assays were performed as described previously [26]. Morphology of the aggregates was characterized by transmission electron and atomic force microscopy. Further details are given in the Supplementary Information.

### Cell viability assays

Cell viability assays were carried out with human neuroblastoma cells (SK-N-SH), embryonic stem cell (ES)-derived neurons and induced pluripotent stem cell (iPSC)-derived neurons. Further details on cultivation and assay procedures are described in the Supplementary Information.

## Results

### Phosphorylation state-specific antibodies detect pSer26Aβ aggregates in transgenic mouse models of AD

**Fig. 1:**
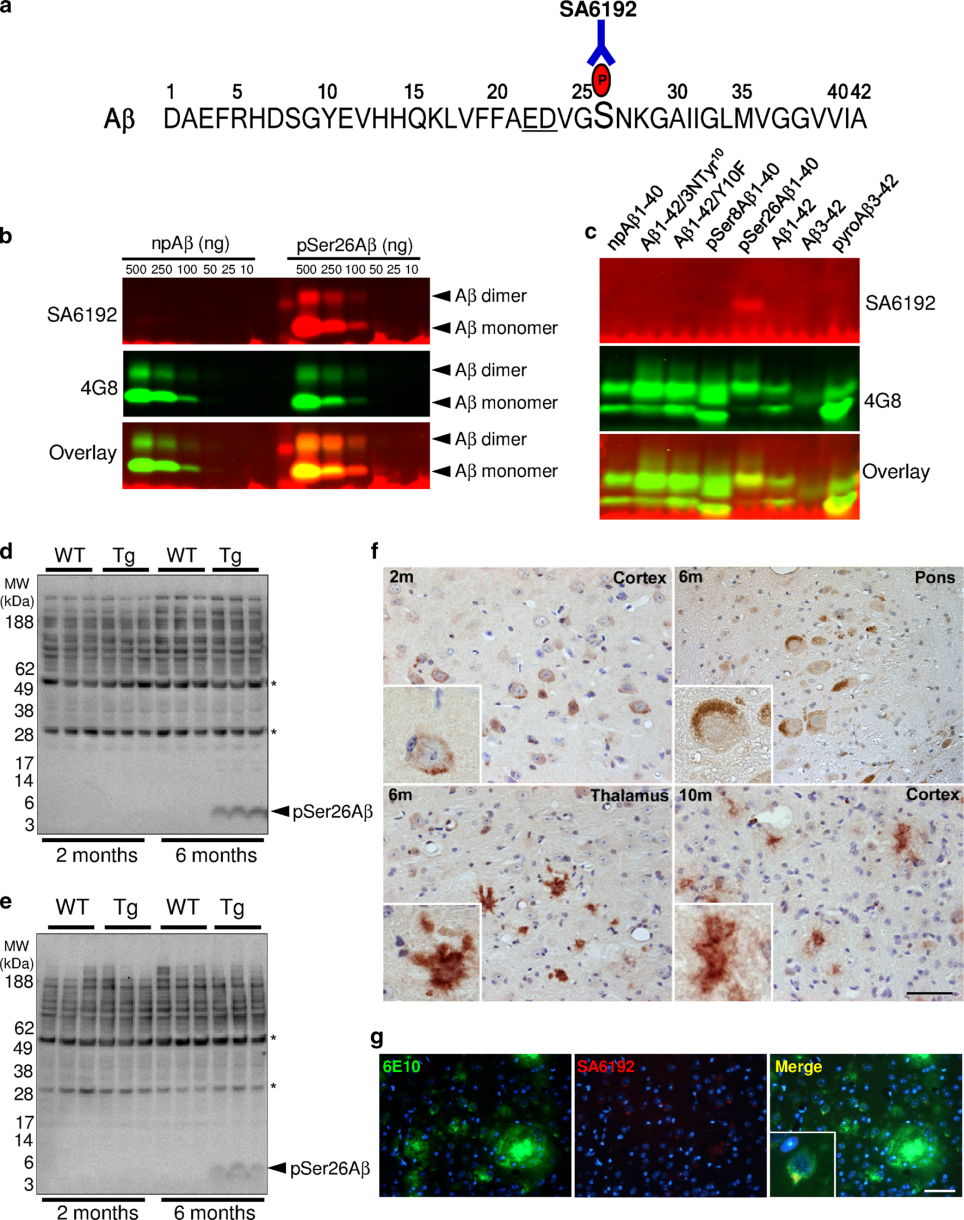
Specific detection of Aβ phosphorylated at Ser26 in transgenic mouse models of AD. **a** Amino acid sequence of human Aβ indicating the phosphorylation site at Ser26. Underlined glutamic acid (*E*) and aspartic acid (*D*) residues comprise a consensus phosphorylation sequence for casein kinase 1. **b**, **c** SA6192 antibody specifically detects pSer26Aβ in immunoblotting without cross-reactivity against other post-translationally modified or non-modified Aβ variants (**c**). Generic 4G8 antibody recognizes non-modified and all the modified Aβ variants (**b**, **c**). **d**, **e** WB analysis of sucrose (**d**) and SDS (**e**) fractions of 2- and 6-months-old APP/PS1KI (Tg) and non-Tg (WT) mouse brain homogenates revealed the presence of pSer26Aβ in vivo. SA6192 showed no reactivity with endogenous mouse APP in non-transgenic mice, further demonstrating the specificity of this antibody (Supplementary Fig. 1a, b). The* bands* indicated by *asterisks* likely represent heavy and light chains of endogenous immunoglobulins. **f** Immunohistochemical staining of 2-, 6-, and 10-month-old APP/PS1KI mouse brain tissues with SA6192 antibody demonstrates the occurrence of intraneuronal (2 and 6 months) and extracellular (10 months) pSer26Aβ deposits in different brain regions. **g** Double-labelling with 6E10 (*green*) and SA6192 (*red*) revealed intraneuronal immunoreactivity of SA6192. The *inset* in the merged image shows a higher magnification of 6E10 and SA6192 co-localization (*yellow*) within the cell body of a cortical neuron. Scale bars **f** and **g** 50 μm

Post-translational modifications could alter the aggregation, degradation and toxicity of Aβ [26–28, 30, 43, 57]. Synthetic Aβ peptides phosphorylated on either Ser8 or Ser26 showed faster formation of oligomeric assemblies in vitro [26, 41]. To specifically investigate Ser26-phosphorylated Aβ (pSer26Aβ) species in vivo, we generated phosphorylation state-specific antibodies (Fig. 1a). Double-affinity purified antibody SA6192 was highly specific for Aβ phosphorylated at Ser26 (Fig. 1b). It did not detect Ser8 phosphorylated (pSer8Aβ), pyroGlu-modified (pyroAβ3–42), N-terminally truncated (Aβ3–42), or nitrosylated (3NTyr10-Aβ) Aβ variants (Fig. 1c), while the generic 4G8 antibody which recognizes an epitope between amino acids 17 and 24 of the Aβ domain, detected npAβ (Fig. 1b) and all the tested peptide variants similarly (Fig. 1c). SA6192 did not detect full-length APP or its C-terminal fragments in brain extracts of transgenic mice, suggesting selective phosphorylation of Ser26 after the generation of Aβ (Supplementary Fig. 1a, b). Detection of pSer26Aβ by the SA6192 antibody was efficiently blocked with synthetic pSer26Aβ, but not with synthetic npAβ peptide, further demonstrating the specificity of this antibody (Supplementary Fig. 1c, d). We took advantage of the SA6192 antibody to characterize the deposition of pSer26Aβ peptides in transgenic mouse brains. Western immunoblot analysis of brain extracts from APP/PS1KI transgenic mice showed the presence of pSer26Aβ peptides in water-soluble (predominantly containing extracellular soluble Aβ) and in SDS-soluble fractions (predominantly containing intracellular and membrane-associated Aβ) at 6 months of age (Fig. 1d, e). pSer26Aβ reactivity was not detected in non-transgenic mouse brains. The quantification of the SA6192 immunoreactivity of water-soluble APP/PS1KI transgenic mouse brain extracts using synthetic pSer26Aβ as standard revealed that ~10–15 % of extracted monomeric Aβ is in a phosphorylated state (50 μg protein from water-soluble brain extracts contained ~0.17 ± 0.03 ng of pSer26Aβ and ~1.55 ± 0.09 ng of total Aβ).

In 2-month-old transgenic mice, pSer26Aβ was not detectable by Western immunoblotting (Fig. 1d, e). Interestingly, immunohistochemistry revealed abundant deposition of pSer26Aβ intraneuronally in 2-month-old animals when extracellular plaques were hardly detectable (Fig. 1f). The pronounced intraneuronal reactivity was also detected in older mice in different brain regions. Occasionally, extracellular deposits were also positive for pSer26Aβ (Fig. 1f). Double-labelling with pSer26Aβ and generic Aβ antibodies specifically demonstrate preferential intraneuronal accumulation of pSer26Aβ in the presence of pronounced extracellular plaques (Fig. 1g). As compared to staining with generic Aβ antibodies, pSer26Aβ reactivity was restricted to structures in the core of the plaques in aged transgenic mouse brains (10 months) (Supplementary Fig. 2a). Double-staining also revealed the association of reactive astrocytes in the vicinity of neurons with intracellular pSer26Aβ (Supplementary Fig. 2b). Reactivity of the SA6192 antibody was not observed in control mice (Supplementary Fig. 3c). SA6192 immunoreactivity in transgenic mice was efficiently blocked by synthetic pSer26Aβ, further supporting the specific detection of pSer26Aβ deposits (Supplementary Fig. 3b, c). Additional immunohistochemical staining of 6- and 12-month-old 5XFAD mouse brains also demonstrated intraneuronal accumulation of pSer26Aβ aggregates and few extracellular pSer26Aβ positive plaques (Supplementary Fig. 4). These data demonstrate a unique pattern of deposition of pSer26Aβ that differs from that of other Aβ species, including post-translationally modified variants like pSer8Aβ [26, 29] or pyroglutaminated Aβ [43, 57].

### Selective intraneuronal deposition of pSer26Aβ in human brains

**Fig. 2:**
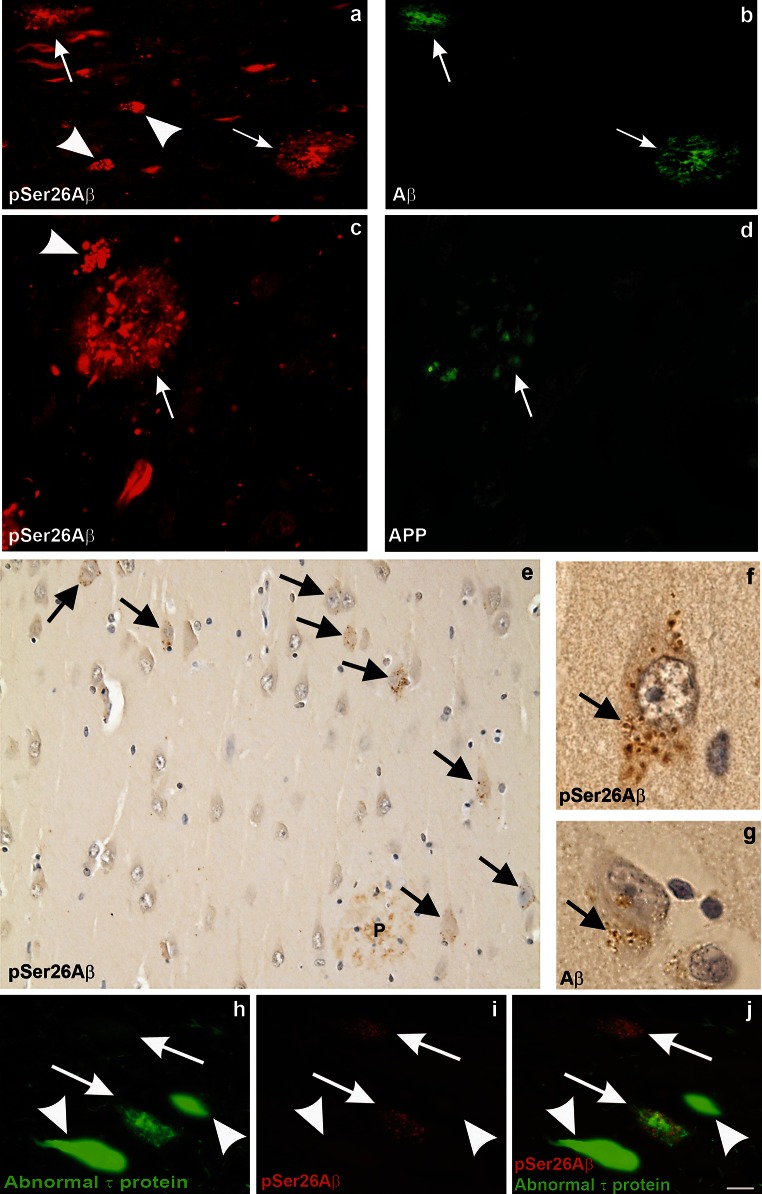
Detection of intraneuronal pSer26Aβ aggregates and GVDs in human AD brains. Detection of pSer26Aβ in extracellular (*arrows*) and intraneuronal (*arrowheads*) deposits in the hippocampal CA1 subfield (**a**, **c**, **e**, **f**, **g**, **i**). The extracellular Aβ plaques are co-stained with anti-Aβ_17–24_ (4G8) (**b**), and anti-APP antibody (22C11) (**d**).The central amyloid core is stained with SA6192 and 4G8 but not with anti-APP indicating the co-deposition of pSer26Aβ together with non-phosphorylated Aβ in plaques (Supplementary Fig. 5a–c). Note the intraneuronal globular aggregates reactivity is selectively observed with pSer26Aβ (*arrowhead* in **a**, **c**), but not with APP antibodies. Immunohistochemical analysis demonstrates strong intraneuronal granular cytoplasmic pSer26Aβ inclusions (*arrows* in **e**), and only weakly stained extracellular pSer26Aβ-positive plaques (*P* in **e**) (Supplementary Fig. 4d). These granular inclusions exhibit the morphological pattern of granulovacuolar degeneration (GVD) and most frequently occur in the CA1-subiculum area of the hippocampal formation (*arrow* in **f**). GVD was also detected by anti-Aβ_17–24_ staining (*arrow* in **g**). pSer26Aβ-positive GVD lesions colocalized with abnormal-phosphorylated τ in neurons (*arrows* in **h**–**j**). Note that neurofibrillary tangles were not labelled with anti-pSer26Aβ antibody (*arrowhead* in **h**–**j**). The *panels* in this figure are representative images from 4 different AD brains (**a**, **b** case # 7; **c**, **d** case # 3, **e**–**g** case # 1 and **h**, **i** case # 5 of supplementary Table 2). *Scale bars* **a** and **b** 50 µm; **c** and **d** 30 µm; **e** 20 µm; **f** and **g** 5 µm; **h**–**j** 20 µm

Human AD brains also revealed a specific accumulation pattern of pSer26Aβ. pSer26Aβ could be detected in individual cored-neuritic plaques and partially overlapped with the pattern of antibodies raised against the middle region of Aβ (4G8; epitope 17–24) (Fig. 2a, b; Supplementary Fig. 5a, b). pSer26Aβ was also detected in APP-positive dystrophic neurites in these plaques (Fig. 2c, d). Anti-pSer26Aβ also stained additional material indicating the deposition of pSer26Aβ within extracellular plaques (Supplementary Fig. 5a, c). A considerable number of diffuse plaques were also stained with anti-pSer26Aβ (Supplementary Fig. 5d). By analysis of the distinct plaque-types occurring in the medial temporal lobe, pSer26Aβ-positive material was restricted to diffuse, cored and neuritic plaques as well as subpial band-like amyloid (Supplementary Fig. 5d), whereas fleecy amyloid and presubicular lake-like amyloid was not stained in the cases studied here (Supplementary Table 1). Moreover, staining of pSer26Aβ in amyloid plaques was restricted to the symptomatic AD cases observed here. No pSer26Aβ-positive plaques are observed in pathologically diagnosed preclinical AD (p-preAD) cases.

Notably, pSer26Aβ was also detected inside of neurons that showed no or only faint reactivity with antibodies against APP or generic Aβ (Fig. 2a–d, arrowhead). The diffuse neuronal staining was not only detected in AD, but also in pathological pre-AD and even in control cases. The intraneuronal pSer26Aβ showed the typical morphological pattern of granulovacuolar degeneration (GVD) [48]. The morphological distribution of the pSer26Aβ-positive granules predominantly in neurons of the CA1-subiculum regions of the hippocampal formation fitted with that known for GVD (Fig. 2e–g) [48]. Interestingly, most neurons with GVD lesions also contained abnormally phosphorylated τ-protein (Fig. 2h–j), phosphorylated transactive response DNA-binding protein (pTDP43) and casein kinase 1δ/ε (Supplementary Fig. 5e–h). Notably, CK1 indeed could phosphorylate Aβ at Ser26 (Supplementary Fig. 6a–d), suggesting a phosphorylation of Aβ by CK1 in GVD compartments. Interestingly, pSer26Aβ was consistently detected together with pTDP43 in GVD, even in p-preAD and non-AD control cases (Supplementary Table 2).

### Peculiar aggregation behaviour of pSer26Aβ

**Fig. 3:**
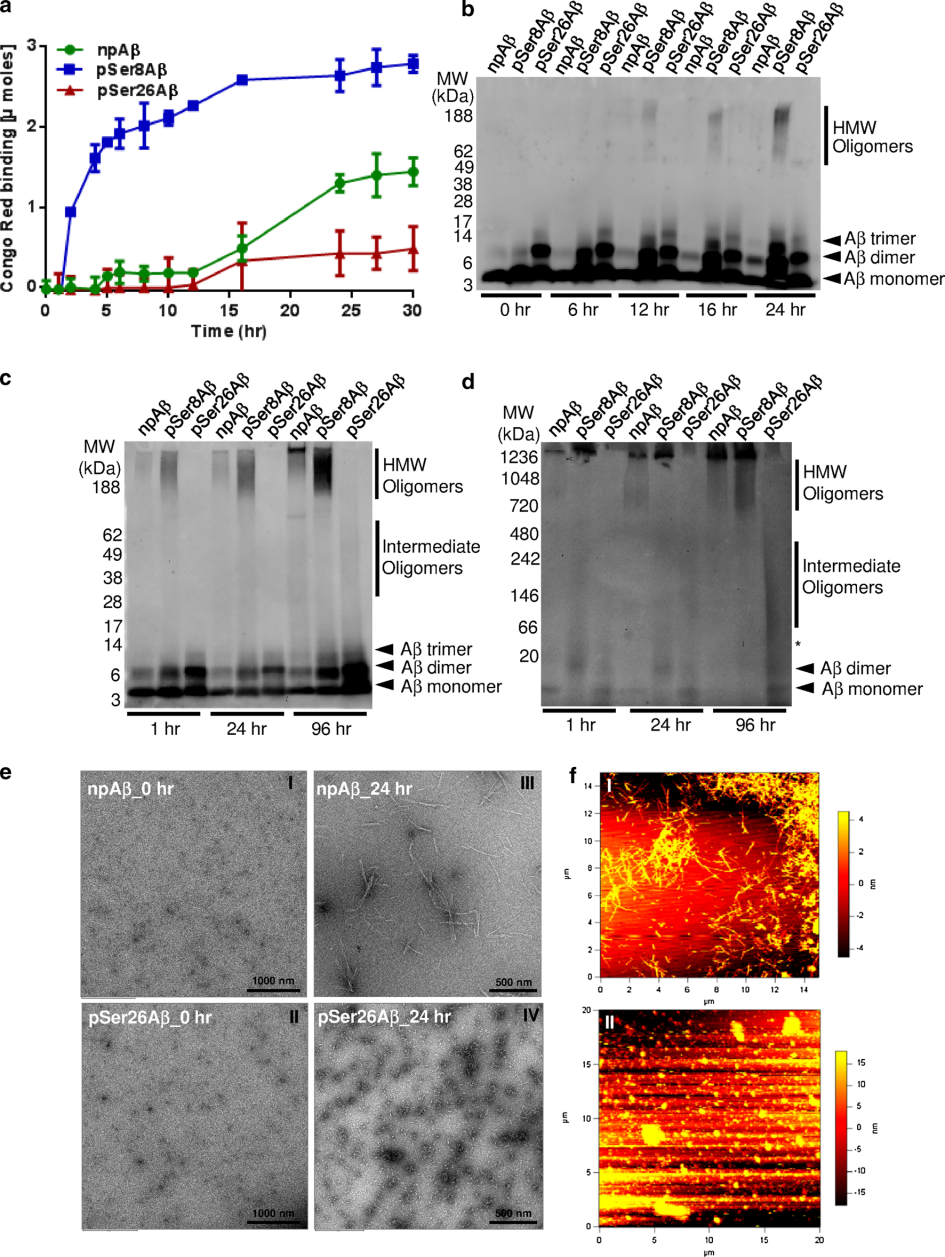
pSer26Aβ selectively forms oligomers without fibril formation. **a** Congo Red (CR) binding assay showing the decreased CR dye binding to pSer26Aβ as compared to npAβ and pSer8Aβ peptides. **b** SDS-PAGE and Western immunoblot detection of Aβ variants after different times of aggregation (see also supplementary Fig. 7). SDS-PAGE (**c**) and native-PAGE (**d**) analysis of the aggregates collected at different incubation time demonstrates the lack of HMW pSer26Aβ assemblies, even after prolonged incubation time (96 h). Monoclonal 82E1 antibody was used for immunoblotting. **e** Transmission electron microscopy (TEM) images demonstrate granular non-aggregated structures of npAβ and pSer26Aβ peptide samples at 0 h (**e** *I*, *II*). After 24 h of incubation, mature fibrils are only seen with npAβ (**e** *III*), whereas pSer26Aβ predominantly shows spherical non-fibrillar chain-like globular structures (**e** *IV*). **f** Atomic force microscopy (AFM) images of npAβ and pSer26Aβ after 24 h of aggregation further confirm the formation of fibrillar aggregates of npAβ (**f, i**) and non-fibrillar globular assemblies of pSer26Aβ peptide (**f** *II*, see also Supplementary Fig. 8)

Phosphorylation of Aβ at Ser26 alters plasticity of a critical turn region and impairs fibrillization [41]. Accordingly, pSer26Aβ showed strongly reduced binding of Congo Red (CR) and Thioflavin T (ThT) as compared to npAβ (Fig. 3a; Supplementary Fig. 7a). In contrast, phosphorylation at Ser8 strongly increased CR and ThT binding. Kinetic analysis revealed a slight but very rapid increase in ThT binding of pSer26Aβ that did not further increase over time (Supplementary Fig. 7a; inset), indicating rapid formation of smaller assemblies without proceeding to fibril formation (Fig. 3b; Supplementary Fig. 7b). In denaturing (Fig. 3c) and non-denaturing PAGE (Fig. 3d), pSer26Aβ was detected as smaller oligomeric assemblies (i.e., dimers and trimers) already at very short incubation periods that persist even after longer incubation time, consistent with a very rapid self-assembly of this Aβ variant. As already indicated by the CR and ThT binding assays, pSer8Aβ reached a higher aggregation state than npAβ represented by the increased reactivity in the upper parts of the gel (Fig. 3b–d; Supplementary Fig. 7b). Even after longer incubation periods, pSer26Aβ only formed intermediate oligomeric forms migrating between 30 and 80 kDa in SDS-containing denaturing gels (Fig. 3c) and between 30 and 400 kDa in native gels (Fig. 3d). npAβ and pSer8Aβ formed higher oligomeric and fibrillar (<1000 kDa) assemblies (Fig. 3c, d). Transmission electron microscopy (TEM) and atomic force microscopy (AFM) revealed only heterogeneous globular species of various sizes of pSer26Aβ without formation of fibrillar structures, as seen with npAβ peptide (Fig. 3e, f; Supplementary Fig. 8).

### Increased toxicity of pSer26Aβ in human neurons

**Fig. 4:**
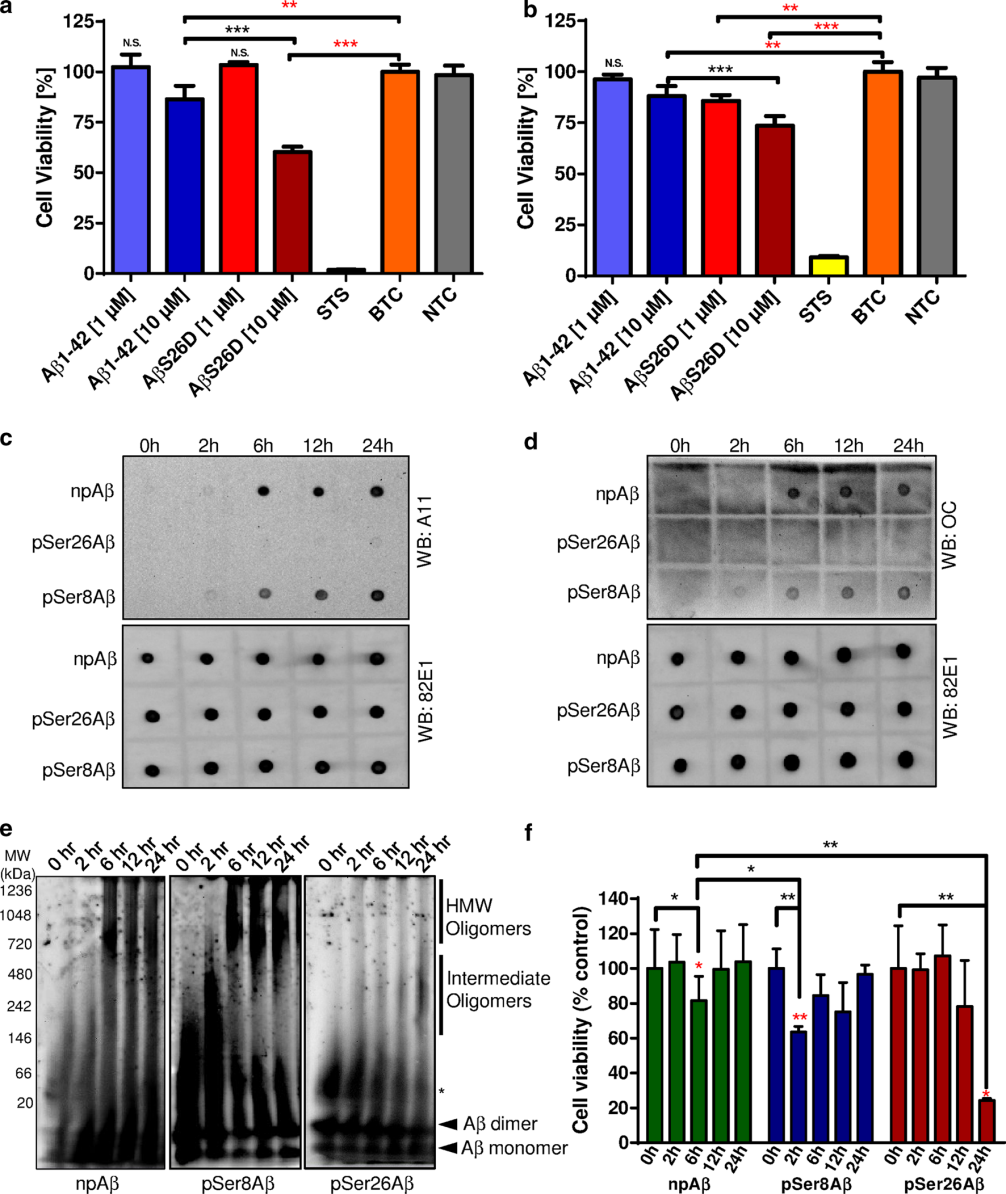
Increased cytotoxicity of pSer26Aβ. Human neuroblastoma cells (**a**) and embryonic stem cell-derived neurons (hESNs) (**b**) were incubated with npAβ (Aβ1–42) and pseudophosphorylated (AβS26D) peptides. Cell viability was analysed by the Presto Blue assay. Both Aβ variants (Aβ1–42 and Aβ26D) did not exert toxicity at lower concentration (1 µM) in neuroblastoma cells. At concentrations of 10 µM, the pseudophosphorylated AβS26D variant was more toxic than the unmodified peptide. (**a**). In hESNs, AβS26D was already toxic at a 1 µM concentration, where unmodified Aβ had no effect. At 10 µM, AβS26D also exterted pronounced toxicity as compared to unmodified Aβ (b). (*p* < 0.01; *red stars* indicate statistical significance of the indicated *bar graphs* versus vehicle controls; *black stars* indicate statistical significance between the indicated *bar graph* pairs; mean ± standard error of the mean (SEM), *n* = 18 replicates from 3 independent experiments). *STS* staurosporine treatment (positive control), *BTC* buffer treatment control (similar volume of PBS without Aβ), *NTC* non-treated control (no addition of PBS to culture media), *N.S.* not significant. **c**, **d** *Dot blotting* of npAβ, pSer8Aβ and pSer26Aβ variants collected at the indicated time periods of incubation (0, 2, 6, 12 and 24 h) with conformation-dependent anti-amyloid oligomer-specific A11 (**c** *upper panel*) and OC (**d** *upper panel*) antibodies. Reprobing of the membranes with generic 82E1 antibody confirms the presence of Aβ variants (**c**, **d** *lower panels*). **e** Native-PAGE of the npAβ, pSer8Aβ and pSer26Aβ samples shows the kinetic differences in formation intermediate and HMW Aβ assemblies. Mouse monoclonal 82E1 antibody was used for probing the blots. *Asterisk* indicates trimeric/tetrameric Aβ assemblies. **f** Aggregates of npAβ, pSer8Aβ and pSer26Aβ were added to induced pluripotent stem cell (iPSC)-derived neurons and incubated for 50 h.The most cytotoxic species observed were the pSer26Aβ aggregates after 24 h of aggregation (*p* < 0.01; *red stars* signify statistical significance of the indicated *bar graphs* versus buffer controls; *black stars* signify statistical significance between the indicated *bar graph* pairs; mean ± SEM)

To assess the toxicity of the pSer26Aβ in a human neuronal model, we used human neuroblastoma cells, human neurons differentiated from embryonic stem cell (ESC) and induced pluripotent stem cell (iPSC)-derived neural stem cells (lt-NES) cells. In a first set of experiments, the different Aβ variants were added without prior aggregation to human neuroblastoma cells (Fig. 4a) and to differentiated neurons (Fig. 4b). As compared to the non-phosphorylated peptide, Aβ pseudophosphorylated at position 26 (AβS26D) induced increased toxicity in neuroblastoma cells (Fig. 4a) and also in hESC-derived neurons (Fig. 4b). Even at concentrations when npAβ showed no overt toxicity, AβS26D impaired neuronal metabolism comparable to the effect of a tenfold higher concentration of npAβ in both neuroblastoma cells (Fig. 4a) and human ESC-derived neurons (Fig. 4b). To specifically assess the toxic properties of different Aβ variants depending on their aggregation state, we next exposed human iPSC-derived neurons to preformed assemblies of npAβ, pSer8Aβ and pSer26Aβ. Dot blot analysis of the different preformed Aβ assemblies showed significant differences in their immunoreactivity against conformation-dependent anti-oligomer antibodies such as A11 (Fig. 4c) and OC (Fig. 4d; Supplementary Fig. 9) [21, 22]. npAβ and pSer8Aβ assemblies were detected by both A11 and OC antibodies after 2–6 h of incubation. However, both antibodies revealed only very little if any reactivity for assemblies formed by pSer26Aβ (Fig. 4c, d). Native-PAGE analysis showed that the samples from different incubation times contained assemblies of different sizes (Fig. 4e). After 2–6 h of aggregation, samples of npAβ and pSer8Aβ contained oligomers of intermediate size (150–480 kDa). After 6–24 h, npAβ and pSer8Aβ also formed high molecular weight assemblies, which were not detected with pSer26Aβ. Instead, oligomers of intermediate size formed by pSer26Aβ were prominently detected at 24 h of incubation. Monomeric and dimeric Aβ were detected at all time points. However, these forms might also result from dissociation of aggregates, even during native-PAGE conditions. Notably, npAβ and pSer8Aβ variants exerted toxicity only at 6 and 2 h of pre-aggregation, respectively, and lost their toxic activity during extended aggregation (Fig. 4e, f). After longer aggregation periods, toxicity of npAβ and pSer8Aβ was decreased. A similar behaviour was previously observed for pyroE3-modified Aβ [39]. Compared to npAβ and pSer8Aβ, pSer26Aβ exerted strongest toxicity (Fig. 4f). Toxicity of pSer26Aβ was observed after 24 h of pre-aggregation. Interestingly, during this time of incubation, pSer26Aβ also formed intermediate-size oligomers of 150–480 kDa, but no larger assemblies or fibrils (Fig. 4e, f).

## Discussion

The present data reveal peculiar characteristics of Ser26 phosphorylated Aβ in aggregation, brain deposition and neurotoxicity. In contrast to non-modified Aβ or other Aβ variants with post-translational modifications in the N-terminal domain of Aβ, including Glu3 pyroglutaminated [43, 57], Ser8 phosphorylated [26, 29], Tyr10 nitrated forms of Aβ [30], pSer26Aβ does not form higher prefibrillar or fibrillar assemblies. Instead, pSer26Aβ forms stable oligomers of intermediate size that exert pronounced toxicity on human neurons.

In many neurodegenerative diseases, soluble oligomers of pathogenic proteins are considered as the principal toxic forms, and the accumulation of large fibrillar deposits may be inert or even protective [1, 4, 15, 19, 47]. Thus, Aβ peptide aggregation into toxic, soluble oligomers is considered as an important event in the pathogenesis of AD [31, 32, 53]. This is also supported by findings with transgenic animal models where pathological changes are frequently observed prior to the onset of amyloid plaque accumulation [5, 16, 49]. In addition, soluble Aβ correlates better with dementia than insoluble fibrillar deposits [1, 11, 31, 32, 52], further suggesting that soluble oligomeric forms of Aβ may represent the primary toxic species in AD pathogenesis. Our results indicate that phosphorylation at Ser26 results in the specific formation of low and intermediate molecular weight, soluble oligomers. These pSer26Aβ oligomers are a persistent structural entity that remain as non-fibrillar assemblies and do not produce high molecular weight Aβ oligomers or fibrils even upon extended incubation time.

Monomeric Aβ is intrinsically disordered in aqueous solution. During conversion into fibrils, two β-strands are formed (residues Val12–Val24 and Ala30–Val40). These two β-strands form parallel β-sheets through intermolecular hydrogen bonding, whereas the intervening region comprising residues Gly25–Gly29 forms a bend-like structure that brings the two β-sheets in contact through sidechain–sidechain interactions [35, 51]. Formation of this turn/bend-like structure from Gly25 to Gly29 is important for fibrillization of Aβ and is one of the earliest events in Aβ self-association and nucleation of Aβ monomers as supported by several experimental and computational studies [2, 24, 35, 38, 41, 51]. Mutations such as the Flemish (A21G), Italian (E22K), Arctic (E22G), Dutch (E22Q), Osaka (E22Δ), and Iowa (D23N) that cause FAD and CAA are localized close to this critical region and interfere with turn formation and fibrillization [8, 10, 14, 18, 24, 25]. Furthermore, computational studies have indicated an interaction of Asp23 and Ser26 that is particularly important in organizing Aβ structure [2]. As Ser26 is located within the Gly25–Gly29 turn motif, phosphorylation of Ser26 in this turn region could play a crucial role in Aβ monomer folding, oligomerization and assembly. Introduction of a negatively charged phosphate group at this position could cause intermolecular repulsive interactions that might lead to destabilization of the fibrillar conformation. The importance of Ser26 is further supported by studies demonstrating that substitution of this residue by proline or cysteine residues alters fibrillization of Aβ [40, 55]. Furthermore, NMR spectroscopy and molecular dynamics simulations have shown that phosphorylation of Ser26 decreases the propensity of Aβ to form a β-hairpin, rigidify the region around the modification site and interfere with formation of a fibril-specific salt bridge between Asp23 and Lys28 [41]. Our present data indicate that phosphorylation at Ser26 promotes the formation of a stable and neurotoxic Aβ assembly, thereby suppressing the formation of larger prefibrillar or fibrillar assemblies with lower toxic activity.

Several studies revealed that intraneuronal accumulation of Aβ precedes its extracellular deposition in AD patients and transgenic mouse brains and correlates with neurodegeneration [5, 16, 23, 29, 56, 58]. Immunohistochemical analysis of the transgenic mouse and human brains demonstrated intracellular accumulation of pSer26Aβ, thereby resembling findings on accumulation of intracellular Aβ oligomers without extracellular plaques in transgenic mice expressing the APPE693Δ mutant [49]. This FAD mutation (Osaka) is located within the Aβ sequence and produces an Aβ variant lacking glutamate-22 (E22Δ) that exhibits enhanced oligomerization without fibrillization [50], very similar to the behaviour of pSer26Aβ. Notably, the intraneuronal pSer26Aβ accumulations in the human AD brain are observed in GVDs. GVDs are one of the pathological hallmarks commonly found in hippocampal pyramidal neurons of patients with aging-related neurodegenerative diseases including AD [48], and defined as electron-dense granules within double membrane-bound cytoplasmic vacuoles present in neurons, having an immunohistochemical signature that suggests that they derive from the autophagic system [12]. GVDs have been shown to present more frequently in AD brains as compared to age-matched controls, and increases during AD pathogenesis [48]. GVDs appear within hippocampal pyramidal neurons in AD when phosphorylated tau begins to aggregate into early-stage neurofibrillary tangles [46], and correlate with vulnerability and neuronal loss [48]. Characterization of GVDs by immunohistochemical methods led to the identification of protein constituents such as tau, pTDP43, together with protein kinases CK1ε and CK1δ [20]. Interestingly, in vitro phosphorylation assays indeed show that CK1 phosphorylates Ser26 of Aβ, indicating that CK1 could also phosphorylate Aβ in vivo. Notably, pSer26Aβ-positive GVDs were also detected in pathologically preclinical AD (p-preAD) and non-AD controls. Thus, it will be intriguing to further analyse the role of intraneuronal pSer26Aβ and progression of AD from pathologically preclinical AD or non-AD to AD stage. It was also suggested that neurons harbouring GVDs with phosphorylated tau accumulation reflect a ‘toxic’ or ‘apoptotic’ alterations in AD [46].

Together, the present data indicate a critical role of Ser26 phosphorylation in Aβ assembly and oligomerization, and its toxic properties. Thus, pSer26Aβ shows similar characteristics as certain Aβ variants with FAD-associated mutations at Ala21, Glu22 and Asp23 [7, 13, 18, 23, 25, 34, 49, 50]. In contrast to these very rare mutations, phosphorylation of Ser26 can occur on wild-type Aβ and was commonly detected in the brains of sporadic human AD cases and several AD mouse models. Thus, pSer26Aβ might be critically involved in the pathogenesis of the most common sporadic late-onset forms of AD.

## Electronic supplementary material

Supplementary material 1 (PDF 1517 kb)
