# Supplementary material for: Phosphorylation of the amyloid β-peptide at Ser26 stabilizes oligomeric assembly and increases neurotoxicity
Source: Acta Neuropathol. 2016 Feb 22;131:525–37. doi: 10.1007/s00401-016-1546-0 (PMC4789232; doi:10.1007/s00401-016-1546-0)
Supplement: Supplementary file 1 — Supplementary material 1 (PDF 1517 kb) [file 401_2016_1546_MOESM1_ESM.pdf]

Phosphorylation of the amyloid β-peptide at Ser26 stabilizes oligomeric assembly and increases neurotoxicity

Sathish Kumar[1], Oliver Wirths[2], Kathrin Stüber[3,4], Patrick Wunderlich[1], Philipp Koch[3,4], Sandra Theil[1], Nasrollah Rezaei-Ghaleh[5], Markus Zweckstetter[5,6,7], Thomas A. Bayer[2], Oliver Brüstle[3,4,8], Dietmar R. Thal[9] and Jochen Walter[1*]

[*]*Correspondence to: Jochen Walter, Department of Neurology, University of Bonn, 53127 Bonn, Germany.*
Tel: +49 228 287 19782; Fax: +49 228 287 14387; Email: Jochen.Walter@ukb.uni-bonn.de.

Supplementary Material includes:

Supplementary Fig. 1-9

Supplementary Tables 1- 2

Supplementary experimental procedures

Supplementary references

Supplementary Fig. 1

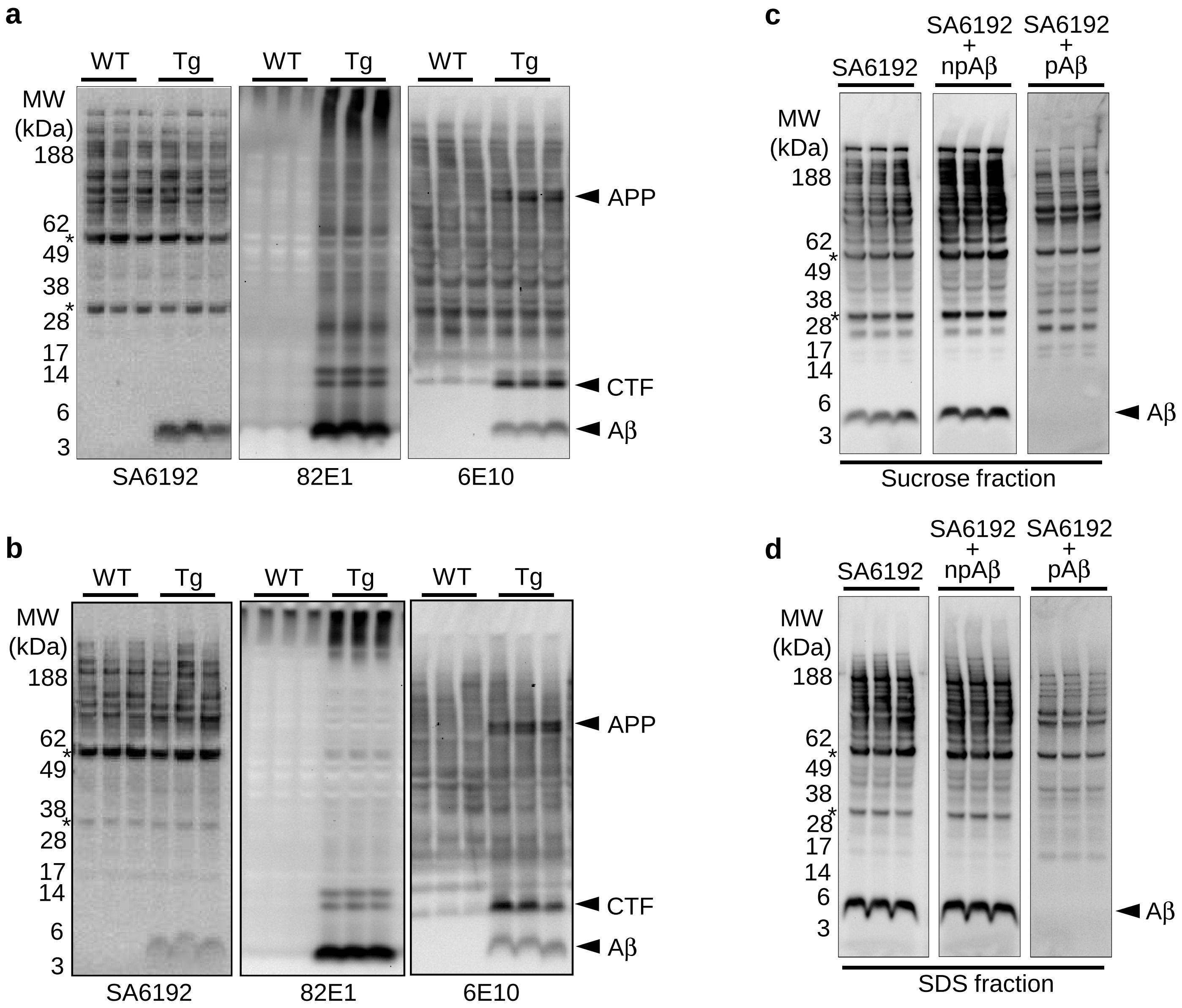

Supplementary Fig. 1: SA6192 antibody specifically recognizes pSer26Aβ peptide in mouse brain homogenates.

a, b) Western blot (WB) analysis of sucrose (**a**) and SDS (**b**) fractions of brain homogenates from non-Tg (WT) and APP/PS1KI transgenic (Tg) mice with SA6192 antibody shows the specific detection of pSer26Aβ peptide. No cross-reactivity is observed against the full-length APP and/or APP-CTFs. In contrast, other commonly used monoclonal antibodies 6E10 and 82E1 detect full-length APP and/or CTFs in addition to Aβ peptide. IgG heavy and light chain reactivity of the antimouse secondary antibody is indicated by asterisks (slightly above 28 kDa and below 62 kDa). **c, d)** WB analysis of sucrose (**c**) and SDS (**d**) fractions of APP/PS1KI mouse brain homogenates with SA6192 antibody alone and in the presence of npAβ or pSer26Aβ peptides. Pre-adsorption with npAβ peptide do not affect SA6192 antibody reactivity. Notably, pre-adsorption of SA6192 antibody with pSer26Aβ peptide completely blocks its reactivity, confirming the antibody specificity. Pre-adsorption of SA6192 with excess amounts of synthetic pSer26Aβ interfere with the reactivity of SA6192 to the bands slightly below 62 kDa in both the sucrose and SDS fractions, and at above 28 kDa in the SDS fraction.

Supplementary Fig. 2

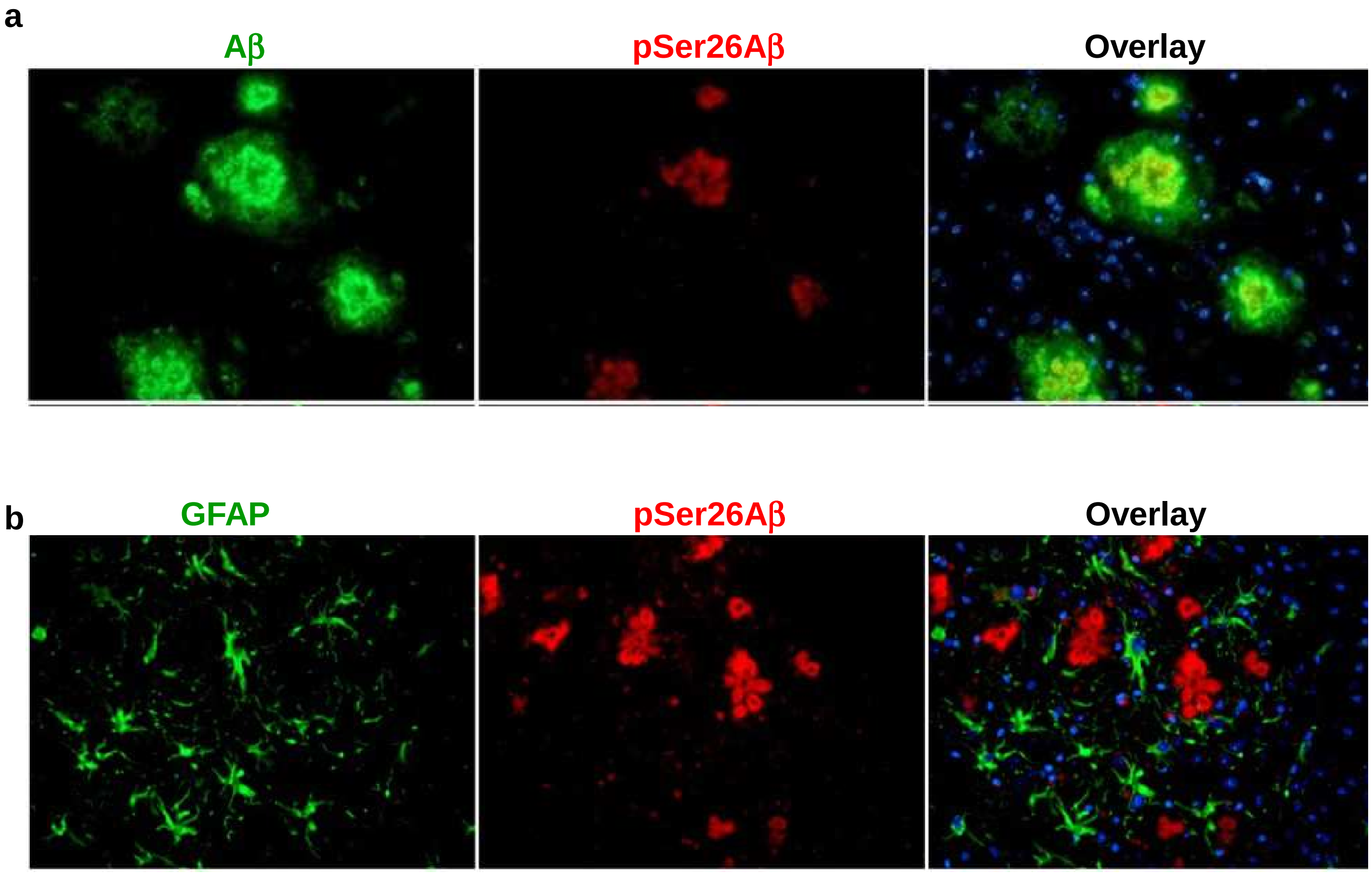

Supplementary Fig. 2: Double-labelling of 10-months-old APP/PS1KI mouse brains.

a) Double labelling with SA6192 and 6E10 antibody shows the preferential deposition of pSer26Aβ deposits in the amyloid core. **b)** Double-staining of pSer26Aβ and the astrocytic marker GFAP shows the association of intraneuronal pSer26Aβ with reactive astrocytes. Images are at 400x magnifications.

Supplementary Fig. 3

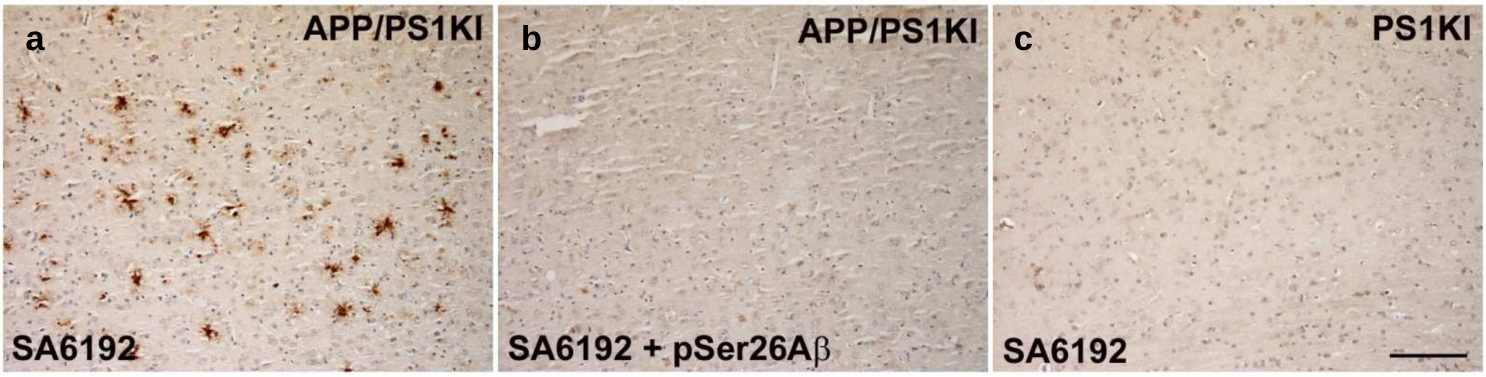

Supplementary Fig. 3: Pre-adsorption of SA6192 antibody with pSer26Aβ peptide blocks immunoreactivity.

a) Immunohistochemical staining of aged APP/PS1KI (13 months) mouse brain sections with SA6192 antibody reveal intraneuronal and extracellular pSer26Aβ immunoreactivity. **b**) Pre-incubation of SA6192 antibody with synthetic pSer26Aβ peptide blocks the SA6192 immunoreactivity. c) SA6192 antibody do not show any staining of plaque-like structures in aged PS1KI (13 months) control animals. *Scale bar* **a**, **b** and **c** 200 µM.

Supplementary Fig. 4

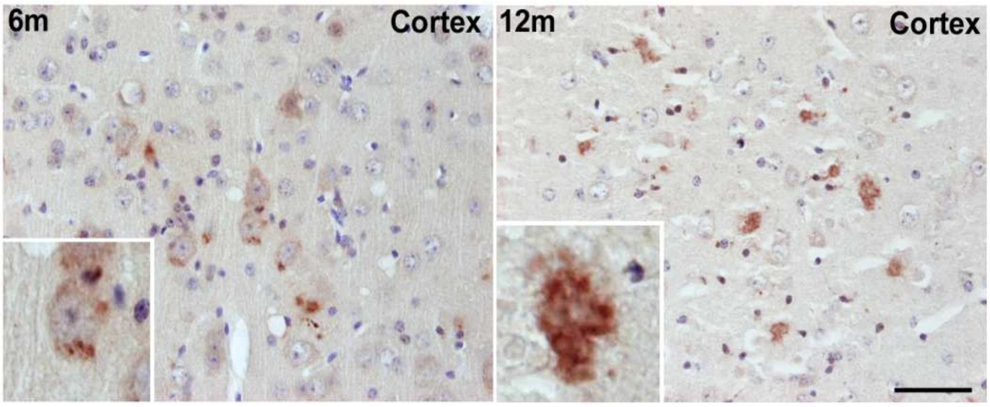

Supplementary Fig. 4: Immunohistochemical detection of pSer26Aβ in 5XFAD mouse brains. Immunohistochemical staining of brain sections from 6- and 12-months-old 5XFAD mice with SA6192 antibody shows the intraneuronal accumulation of pSer26Aβ at 6-months of age. Few extracellular pSer26Aβ deposits are seen at 12-months. *Scale bar* 50 µM.

Supplementary Fig. 5

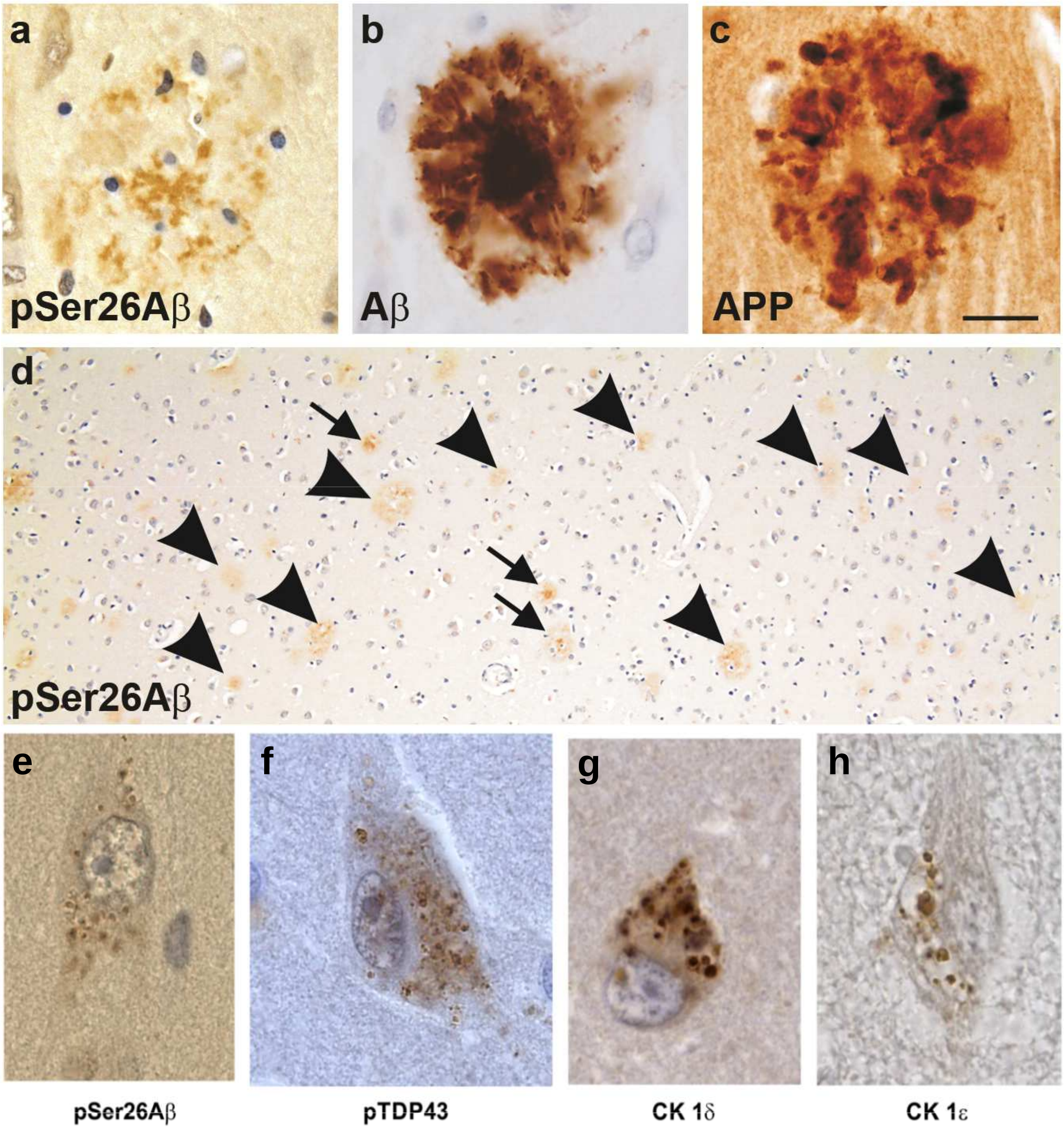

Supplementary Fig. 5: Immunohistochemical detection of pSer26Aβ and GVDs in human AD brain (Case # 1 (Suppl. Tab. 2)).

In plaques of the CA1 region the central amyloid core is stained with anti-pSer26Aβ (**a**) and anti-$A\beta_{17\text{-}24}$ (4G8) (**b**) but not with anti-APP (**c**) indicating that pSer26Aβ deposits together with non-phosphorylated Aβ in plaques. **d)** Diffuse plaques (marked in arrowheads) in the temporal neocortex (Brodmann area 36) can also contain pSer26Aβ in addition to cored plaques (indicated by arrows). **e-h)** GVD lesions in the subiculum-CA1 region were detected with anti-pSer26Aβ (**e**), anti-pTDP-43 (**f**), anti-CK1δ (**g**) and anti-CK1ε (**h**) antibodies.

Supplementary Fig. 6

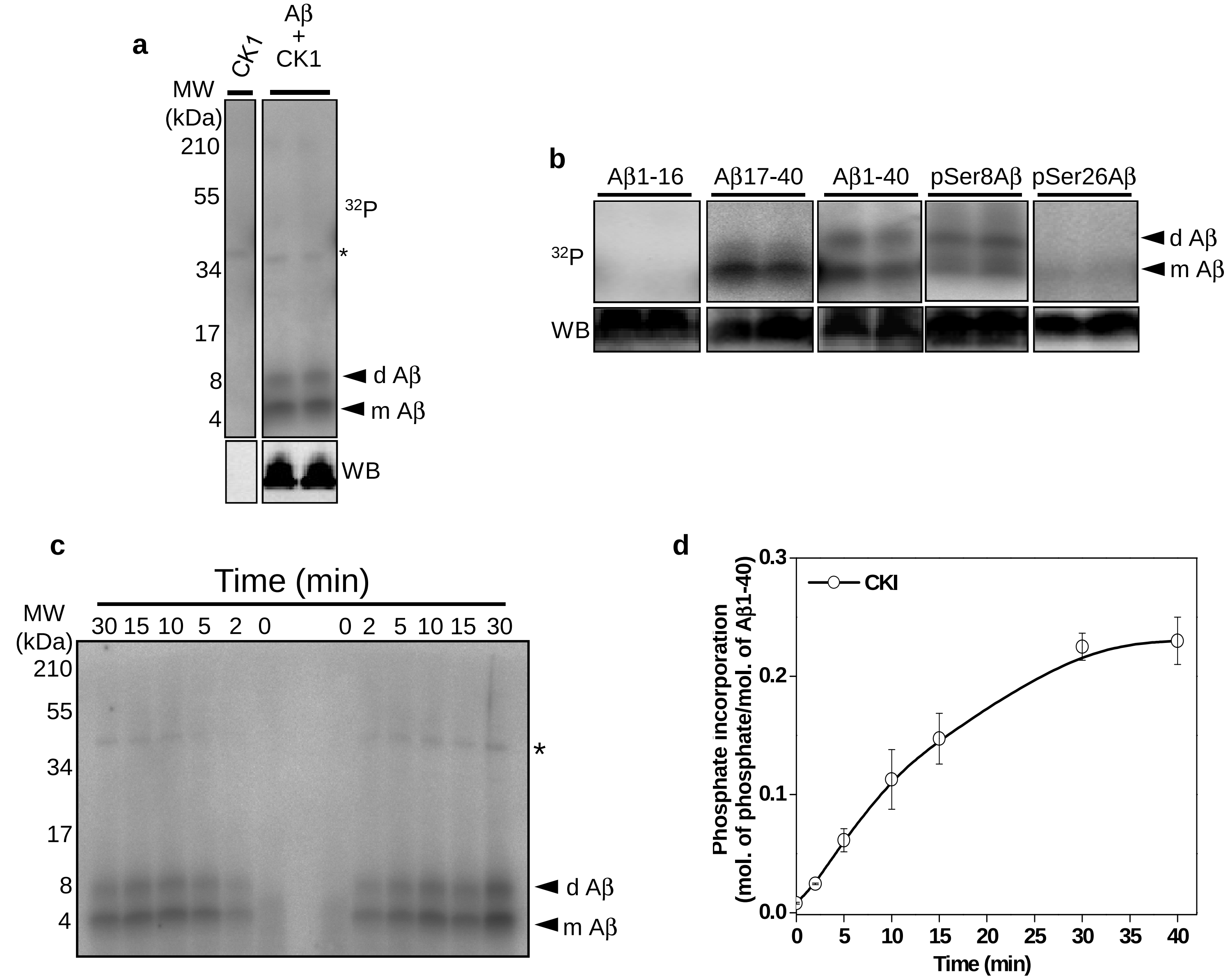

Supplementary Fig. 6: Casein Kinase 1(CK1) phosphorylates Serine-26 residue of Aβ.

a) *In vitro* phosphorylation of Aβ using radiolabelled [γ^{32}P]ATP and catalytic subunits of recombinant CK1 in the presence and absence of Aβ peptide. Autoradiograph (^{32}P) demonstrates the phosphate incorporation. Western blot (WB) confirms the presence of Aβ peptide. Asterisk indicates the autophosphorylation signals of catalytic subunit of CK1. **b)** *In vitro* phosphorylation employing different Aβ variants (Aβ1-16, Aβ17-40, full-length Aβ1-40 and pre-phosphorylated Aβ such as pSer8Aβ and pSer26Aβ confirming the specificity of CK1 phosphorylation at Ser26 residue. Phosphate incorporation can be seen only with Aβ17-40, Aβ1-40, and pSer8Aβ peptide variants. No phosphate is incorporated with Aβ1-16 and pre-phosphorylated pSer26Aβ peptide. The lower panel (WB) shows the presence of Aβ. **c, d)** *In vitro* phosphorylation of Aβ using radiolabelled [γ^{32}P]ATP and catalytic subunit of recombinant CK1 at 32°C for different time intervals as indicated. Autoradiography (^{32}P) showing the phosphorylation of Aβ. **d)** Quantification of the experiment presented in panel (**c**). Values represent mean ± standard deviation (n=4 from 2 independent experiments).

Supplementary Fig. 7

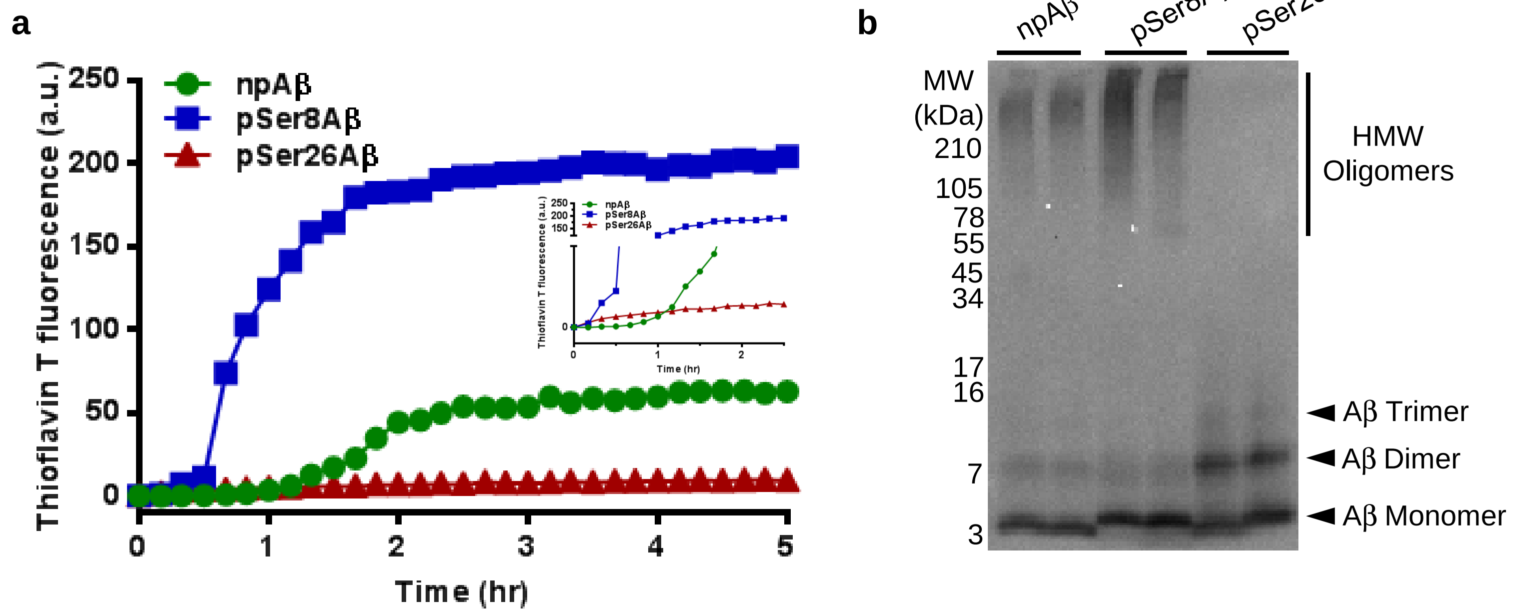

Supplementary Fig. 7: Aggregation of pSer26Aβ in comparison to pSer8Aβ and npAβ.

a) Thioflavin T (ThT) binding assay showing reduced ThT fluorescence to pSer26Aβ as compared to npAβ and pSer8Aβ. Each point represents the mean ± standard deviation (n=4). pSer26Aβ aggregates rapidly as compared to npAβ and pSer8Aβ peptides, indicating a rapid oligomerization (see inset). Despite pSer26Aβ showed a higher ThT fluorescence at the beginning, the fluorescence intensity was not increased further with incubation time, suggesting a rapid formation of smaller assemblies without proceeding to fibrils (see inset). **b**) Aliquots of aggregates collected at the end of ThT assay were separated by SDS-PAGE and immunoblotted with 82E1 antibody. pSer26Aβ produces smaller oligomeric assemblies only (Aβ dimers and trimers). SDS-resistant high oligomeric assemblies are observed only with npAβ and pSer8Aβ peptides. HMW Oligomers- High Molecular Weight Oligomers.

Supplementary Fig. 8

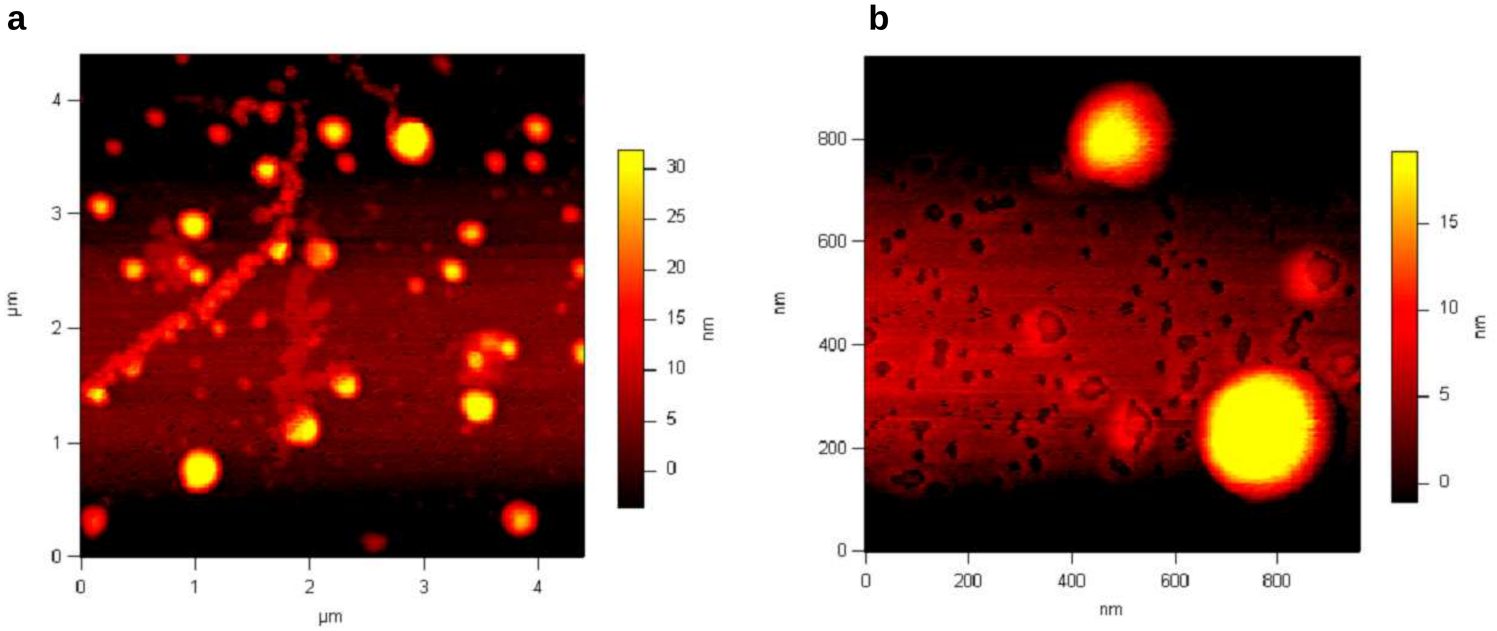

Supplementary Fig. 8: AFM images of pSer26Aβ globular oligomers.

a, b) AFM images of pSer26Aβ oligomers after 72 hours of aggregation. pSer26Aβ failed to form amyloid fibrils even after longer incubation (3 days). pSer26Aβ oligomers remain as globular in nature. Image **b** showing pSer26Aβ globular oligomers at higher magnification.

Supplementary Fig. 9

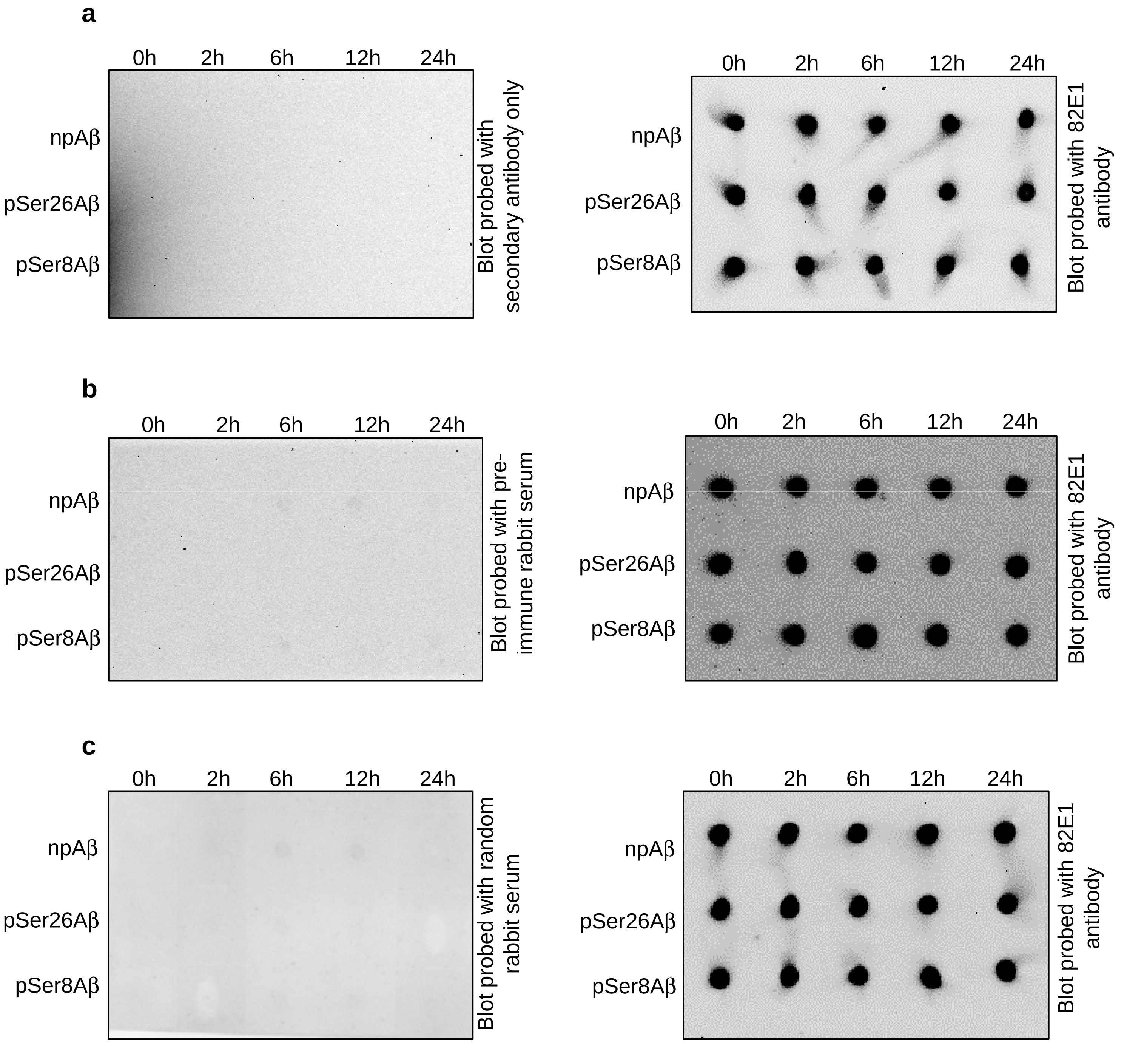

Supplementary Fig. 9: Control of A11 and OC antibody in dot-blots, related to Figure 4.

Dot blots of the oligomerized npAβ, pSer8Aβ and pSer26Aβ variants collected at different time intervals (0, 2, 6, 12 and 24 hr) were probed with secondary anti-rabbit antibody only (**a**), rabbit pre-immune serum (**b**) and unrelated rabbit serum (**c**) (left panels). No reactivity was observed for all the three Aβ variants, with these control conditions. Reprobing of the membranes with generic 82E1 antibody confirms the presence of npAβ, pSer8Aβ and pSer26Aβ variants (right panels).

Supplementary Table 1: pSer26Aβ in AD-lesions.

**Lesion**	**abnormal phosphorylated τ-protein**	**Aβ**	**pSer26Aβ**
Diffuse plaques			
Diffuse non-neuritic plaques	-	+	-/+
Diffuse neuritic plaques	+	+	+
Fleecy amyloid	-	+	-
Subpial band-like amyloid	-	+	-
Presubicular lake-like amyloid	-	+	-
Cored plaques			
Cored non-neuritic plaques	-	+	+/-
Cored neuric plaques	+	+	+
White matter plaques	-	+	-
Neurofibrillary tangles	+	-*	-*
Granulovacuolar degeneration	+	-	+

* Ghost tangles can be associated with Aβ deposits

Supplementary Table 2: List of human cases

Case No.	Age/years	Sex	AβMTL	Braak-NFT	CERAD	NIA-AA	B-Aβ-plaque stage	GVD-stage	Neuropathological diagnosis	pSer26Aβ Pathology
1	81	f	4	5	1	2	3	5	AD	CP, DP, S-bl-Amyloid, GVD
2	83	m	4	5	2	3	3	5	AD	CP, DP, S-bl-Amyloid, GVD
3	86	f	4	6	2	3	3	3	AD	CP, DP, GVD
4	83	m	4	4	3	2	3	5	AD	CP, DP, S-bl-Amyloid, GVD
5	78	f	4	5	2	3	3	5	AD	CP, DP, S-bl-Amyloid, GVD
6	87	f	4	4	1	2	3	3	AD	CP, DP, GVD
7	72	m	2	3	0	1	2	3	p-preAD	GVD
8	66	f	2	2	0	1	2	0*	p-preAD	-
9	83	m	2	3	0	1	1	0*	p-preAD	-
10	66	f	0	0	0	0	0	0	not AD	-
11	62	m	0	0	0	0	0	0	not AD	-
12	69	f	0	0	0	0	0	1	not AD	GVD
13	61	m	0	0	0	0	0	0	not AD	-

CP: Cored plaque, DP:Diffused plaque, S-bl-Amyloid: subpial band-like amyloid, GVD-Granulovacuolar degeneration

Supplementary experimental procedures:

Electrophoresis and Western-blotting

Synthetic peptides or mouse brain extracts were separated on 4-12% NuPAGE Bis-Tris mini or midi gels and transferred onto nitrocellulose membranes. The membrane was incubated sequentially with the indicated primary and respective secondary antibodies and then developed using Odyssey infrared imaging system (LI-COR® Biosciences) employing IRDye® secondary antibodies and/or ECL imager (BioRad Inc) using enhanced chemiluminescence reagents. Quantification was performed by using Image Studio 3.1 (LI-COR® Biosciences) or densitometry analysis using Quantity One software (BioRad Inc).

Biochemical and immunohistochemical analysis of pSer26Aβ in transgenic mouse brains

Extraction of soluble and insoluble Aβ from mouse brains

Brains of APP/PS1KI female bigenic mice or wild type mice were collected. All animals were handled according to German guidelines for animal care.One hemisphere was snap-frozen in liquid nitrogen for biochemical analysis, and the other hemisphere was fixed in 4% paraformaldehyde for immunohistochemistry. The brain homogenates were prepared using buffers containing sucrose and SDS. We weighed frozen mouse hemi brains, added freshly prepared ice-cold 0.32 M sucrose in 50 mM Tris buffer (pH 7.3) containing complete protease and phosphatase inhibitors (Roche Diagnostics, Germany) to the frozen hemi brains at 5:1ratio (Sucrose solution volume / brain wet weight) and homogenized with 20 strokes at a setting of 70 on a mechanical Dounce homogenizer (Brandelin Sonopuls, Germany). Homogenates were then centrifuged at 16,100 g and 4 °C in a HL116 rotor (Eppendorf, Hamburg, Germany) for 30 minutes. After centrifugation, we aliquoted and then stored the supernatant (called sucrose fraction) at -80 °C. The pellet was re-homogenized (5:1 vol/wt) in 50 mM Tris buffer (pH 7.3) containing 2% SDS plus complete protease and phosphatase inhibitors followed by sonification and spun as above. The resultant supernatant (called SDS fraction) was aliquoted and stored at -80 °C until further use. The total protein concentration

in the sucrose and SDS fractions of brain homogenates was determined by BCA™ protein assay kit (Thermo Scientific, USA). For SDS-PAGE analysis, aliquots of sucrose and SDS fractions containing total protein equivalent to 50µg was used. The blots were blocked in 3% BSA (diluted in 1x TBS/T) for 2 hours. Proteins were detected with the indicated primary antibodies and respective horseradish peroxidase-conjugated secondary antibodies using enhanced chemiluminescence (ECL reagent, GE Healthcare). Quantification was done with an ECL imager (BioRad Inc.) and the Quantity One software (BioRad).Western blotting of full-length amyloid precursor protein (Fl-APP), APP C-terminal fragments (APP-CTFs) and Aβ was performed as described previously (Kumar et al., 2011, Kumar et al., 2013).

Immunohistochemistry: In brief, following deparaffinization in xylene and rehydration in a series of ethanol, treatment with 0.3% H_2O_2 in PBS was used to block endogenous peroxidases. Antigen retrieval was achieved by boiling sections in 0.01 M citrate buffer pH 6.0, followed by 3 min incubation in 88% formic acid. Skim milk and fetal calf serum in PBS were used to block non-specific binding sites prior to the addition of primary antibody SA6192 (1:50 – 1:100). The primary antibody was incubated overnight in a humid chamber at room temperature followed by incubation with a biotinylated secondary anti-rabbit antibody (DAKO, Glostrup, Denmark). Staining was visualized using the ABC method using a Vectastain kit (Vector Laboratories, Burlingame, USA) and diaminobenzidine (DAB) as chromogen providing a reddish-brown color. Counterstaining was carried out with hematoxylin.Double-immunofluorescence staining were performed using SA6192 (1:50), 6E10 (1:1000) and GFAP (1:1000, #173011, Synaptic Systems, Goettingen). For detection, secondary anti-mouse and anti-rabbit conjugated 594 DyLight and 488 DyLight antibodies were used.

Immunohistochemistry of human AD brain

Paraffin sections from the human medial lobe were stained with rabbit polyclonal pSer26Aβ-specific SA6192 antibody, mouse monoclonal 4G8 (anti-Aβ17–24) and 22C11 (anti-APP) antibodies. The primary antibodies were detected with biotinylated anti-rabbit IgG secondary antibodies and visualized with avidin-biotin-complex (ABC-Kit, Vector Laboratories) and diaminobenzidine-HCl (DAB). The sections were counterstained with haematoxylin. Double-label immunofluorescence was performed to demonstrate the co-localization of pSer26Aβ with APP and phosphorylated tau. Immunostaining was performed using SA6192 (anti-pSer26Aβ), 4G8 (anti-Aβ17–24), 22C11 (anti-APP) and AT8 (anti-phosphotau) antibodies. The primary antibodies were visualized with carbocyanin 2 (Cy2)-labelled antibodies directed against mouse IgG and Cy3-labelled antibodies against rabbit IgG. These sections were mounted in Corbit without counterstaining. Immunolabelled sections were analyzed with the Leica Microsystems (Wetzlar, Germany) DMLB microscope.

Aβ aggregation assays

Synthetic npAβ, pSer8Aβ and pSer26Aβ peptide stock solution (230 µM) was diluted with 50 mM of sodium phosphate buffer (pH 7.4, 50 mM NaCl, 20 µM ThT and 0.01% sodium azide) to a final Aβ concentration of 100 µM and incubated at 37 °C with gentle stirring. Kinetic of Aβ aggregation was determined by measuring ThT emission intensity using Varian fluorescence spectrophotometer. Excitation and emission wavelengths were 446 and 482 nm, respectively, with slits widths of 10 nm. For CR binding assay, stock solutions of npAβ, pSer8Aβ and pSer26Aβ were diluted to a final concentration of 100 µM in 10 mM phosphate buffered saline solution (PBS, pH, 7.4; supplemented with 0.01% sodium azide), and incubated at 37 °C with gentle stirring. Sample aliquots collected at different time points during aggregation. Later the samples were mixed with 10 µM CR in PBS and incubated for 15 min at room temperature. After incubation, the absorbances were read at 540 nm and 480

nm using a spectrophotometer (Varian Inc.). The amount of bound Congo red was calculated as described previously [5].

Transmission electron microscopy

Wild-type and pSer26Aβ peptide variants solubilised in 50 mM of sodium phosphate buffer (pH 7.4, 50 mM NaCl, 20 µM ThT and 0.01% sodium azide) were incubated at 37 °C with gentle stirring. Aliquots of samples were collected at the beginning (0 hr) and end (24 hr) of the aggregation assays. Samples were diluted, deposited onto carbon-coated copper mesh grids and negatively stained with 2 % (w/v) uranyl acetate. The excess stain was washed away, and the sample grids were allowed to air-dry. The samples were then viewed with a 120-kV transmission electron microscope.

Atomic Force Microscopy

Aliquots of wild-type and pSer26Aβ peptide samples collected at the beginning (0 hr) and end (24 hr) of the aggregation assays (as mentioned before), and allowed to adsorb onto the surface of freshly cleaved mica cover slips. After 10 min, the surface was washed with water and dried three times. Atomic Force Microscopy (AFM) imaging was performed in tapping mode using a MFP-3D AFM machine (Asylum Research, Santa Barbara, CA, USA). To further analyze the morphology of pSer26Aβ aggregates with aging, 50 µl of pSer26Aβ peptide solution (concentration: 100 µM) which had been incubated for 72 hours in the aggregation condition, were AFM imaged as mentioned before.

Cell viability assays

Human neuroblastoma cells (SK-N-SH) were maintained in DMEM supplemented with 10% fetal calf serum (FCS), 100 U/ml penicillin, 100 µg/ml streptomycin and 300 µg/ml glutamine. Presto Blue (PB) cell viability reagent (Life Technologies) was used according to the manufacturer's protocol. SK-N-SH cells in suspension were seeded at 3 x 10^6 cells/well

plated in a 24-well microtitre plate and allowed to attach overnight. Cells were then exposed to various concentrations of npAβ (WT) and phospho-mimicking (pseudophosphorylated) AβS26D Aβ variants (1µM and 10µM), staurosporine (500 nM) or PBS for 72 hours at 37 °C and 5% CO_2 in a humidified atmosphere. After 72 h-treatment, the cells were incubated with 10 µl of PB reagent. Cell viability was analyzed using, fluorescence spectroscopy. The fluorescence absorbance was read with an excitation wavelength at 570 nm and emission wavelengths at 610 nm. Substracted the average fluorescence values of the no-cell control wells from the fluorescence value of each experimental well. To test the Aβ toxicity on human neurons, human embryonic stem lt-NES cells were differentiated for four weeks. Differentiated neurons were then exposed with non-phosphorylated, pseudophosphorylated Aβ variants (Aβ26D) for 72 hours. Staurosporine and PBS were used as controls. After 72 h-treatment, cell viabilitywas measured using a fluorescence plate reader as mentioned above.

For quantitation of cell viability in induced pluripotent stem cell (iPSC)-derived neurons, iPSC-derived neurons were grown in black-walled 96-well tissue culture plates (Corning) were assayed using PB as mentioned above. Aβ oligomers were prepared by dissolving the lyophilized synthetic Aβ (npAβ, pSerAβ and pSer26Aβ variants) in DMSO and incubation at 37 °C with stirring. Aggregates were collected at the indicated incubation times (0, 2, 6, 12 and 24 hours). Aβ oligomers were added to minimal supplemental cell culture medium (114 mM NaCl, 0.219% $NaHCO_3$, 5.292 mM KCl, 1 mM $MgCl_2$, 2 mM $CaCl_2$, 10 mM HEPES, 1 mM glycine, 30 mM glucose, 0.5 mM sodium pyruvate, 0.1% phenol red, 10% NGM). Peptide-containing minimal media were added to cultures to achieve the noted final concentrations of total Aβ. Complete cell death was induced by addition of 500 nM staurosporine. Metabolic activity was measured by Presto Blue® Assay. Unpaired, two-tailed t-tests were used for statistical analysis.

Cultivation and differentiation of human pluripotent stem cell-derived neuronal cultures

Embryonic stem cells (ES cells) [3] or induced pluripotent stem cell (IPS cells)-derived neuronal cultures [4] were generated from long-term self-renewing neuroepithelial stem cells (lt-NES cells). Lt-Nes cells were grown on poly-L-ornithin/laminin coated dishes in Dulbecco's modified Eagle's medium (DMEM)/F12 (Life Technologies), N2 supplement (PAA), 1.6 g/l glucose, B27 (1:1000; Life Technologies), fibroblast growth factor 2 (FGF2) and epidermal growth factor (EGF; both10 ng/ml; R&D Systems) and were splitted 1:2 every 3-4 days. To initiate neuronal cultures, growth factors were withdrawn Neuronal Growth Media (NGM) composed of DMEM/F12 and MACS Neuro Medium (Miltenyibiotec) mixed at 1:1 ratio and supplemented with 1:200 N2 supplement, 1:100 B27 supplement and 300 ng/mL cAMP. 5µM of N-[N-(3,5-Difluorophenacetyl)-L-alanyl]-S-phenylglycine t-butyl ester (DAPT) was added to the media for one week to accelerate differentiation. Cells were then transferred onto Geltrex-coated dishes and further differentiated for 2 weeks. Treatment of neuronal cultures with different variants of Aβ was performed in Minimal Medium as described above for 60h with IPS-cell derived neurons and in NGM for 72h with ES-cell derived neurons.

Peptide competition assay

Peptide competition assay was performed by incubating SA6192 antibody solution with or without 200 molar excess of synthetic npAβ and pSer26Aβ peptide at 4 °C overnight on a rotoshaker. Antibody solutions were centrifuged at 4,000 rpm for 10 min to pellet immune complexes. Supernatant was collected and used for western-blotting analysis.

For immunohistochemistry, immunoabsorption of SA6192 was carried using pSer26Aβ (2µg peptide per 10µl of antibody). Following incubation at room temperature with continuous agitation for 5h, a centrifugation step at 14,000 g for 5 min was carried out and the

supernatant was used for immunostaining. Aged APP/PS1KI mice (age: 13 months) were stained with either SA6192 (1:50) or immuno-absorbed SA6192 antibody.

Dot blotting of Aβ

Aggregates of npAβ, pSer8Aβ and pSer26Aβ were prepared by incubating the synthetic Aβ peptides (concentration: 80 µM) at 37 °C to different incubation times (0, 2, 6, 12 and 24 hours) in an aggregation prone conditions. 2 µl of sample aliquots collected at various time points during aggregation were spotted onto nitrocellulose membranes and subjected to immunoblotting. The membranes were blocked in 5% milk in Tris-buffered saline containing 0.025% Tween 20 (TBS/T) and incubated in rabbit polyclonal A11 [2] and OC [1] primary antibody solutions (4°C, overnight). Membranes were washed with TBS/T followed by incubation with anti-rabbit horseradish peroxidase-conjugated secondary antibodies. The membranes were developed electrochemiluminescecne reagents using Biorad ECL imager. Specific immunoreactivity of the rabbit polyclonal A11 and OC antibodies to npAβ, pSer8Aβ and pSer26Aβ aggregates were verified by performing the control-dot blot experiment. 2 µl of npAβ, pSer8Aβ and pSer26Aβ aggregates were spotted onto nitrocellulose membranes and were subjected to immunoblotting. The membranes were blocked in 5% milk in Tris-buffered saline containing 0.025% Tween 20 (TBS/T) and incubated in seconary anti-rabbit HRP antibody only, random rabbit serum and pre-immune rabbit serum (at 4°C, for overnight). After overnight incubation, membranes were washed with TBS/T followed by the addition of anti-rabbit horseradish peroxidase-conjugated secondary antibodies. The membranes were developed with electrochemiluminescecne reagents using Biorad ECL imager.

***In vitro* phosphorylation of Aβ**

Casein Kinase (CK1) was purchased from New England Biolabs. *In vitro* phosphorylation assays with recombinant CK1 were carried out by incubating 1 µl of Aβ1-40 solution (Stock:

1 mg/ml) in 28 µl of 1x CK1 reaction buffer (50 mM Tris-HCl, 10 mM $MgCl_2$, 5 mM dithiothreitol, pH 7.5) containing 1 µl of 1:25 diluted CK1 (specific activity 2,000,000 units/mg). Phosphorylation reactions were started by the addition of [γ-^{32}P]ATP (end concentration: 10 µM) and allowed to proceed for respective time intervals at 32 °C. Reactions were stopped by the addition of 5x SDS sample buffer and boiling for 5 min. Samples were SDS electrophoresed and western blotted. The membranes were exposed to phosphor imager screen.

Blue native PAGE Electrophoresis

Blue native PAGE was performed according to manufacturer protocol. NativePAGE™ Novex™ 4-16% Bis-Tris precast polyacrylamide gels, respective running buffer, sample preparation buffer and NativeMark unstained protein standard were procured from ThermoFisher Scientific. Detergent free samples were prepared by adding Native-PAGE sample buffer to the synthetic Aβ aggregate mixture which were collected at various incubation times during aggregation from npAβ, pSer8Aβ and pSer8Aβ variants. The samples were mixed well and were electrophoresed without boiling. Electrophoresis was performed in the cold room using pre-chilled running buffers. Gels were run at 150V constant for 2 hours using "Light blue" cathode buffer. After electrophoresis, the gels were carefully removed and blotted onto methanol-activated PVDF membranes employing NUPAGE transfer buffer. After transfer, the membranes were blocked with 5% mild powder (in 1x TBS/T) for 2 hours. After blocking, the PVDF membranes were incubated in mouse monoclonal 82E1 antibody solution at 4 °C for overnight. Membranes were washed with 1x TBS/T (4 x times; 5 minutes interval) and incubated with anti-mouse HRP conjugated secondary antibodies at room temperature for 2 hours. Membraes were washed thoroughly in 1x TBS/T (4 x times; 5 minutes interval) and signals detected by chemiluminescence using Bio-rad Imager.

Supplementary References:

1. Kayed R, Head E, Sarsoza F, Saing T, Cotman CW, Necula M, et al. (2007) Fibril specific, conformation dependent antibodies recognize a generic epitope common to amyloid fibrils and fibrillar oligomers that is absent in prefibrillar oligomers. Mol Neurodegener. 2:18.

2. Kayed R, Head E, Thompson JL, McIntire TM, Milton SC, Cotman CW, et al. (2003) Common structure of soluble amyloid oligomers implies common mechanism of pathogenesis. Science 300: 486-9.

3. Koch P, Opitz T, Steinbeck JA, Ladewig J, Brustle O (2009) A rosette-type, self-renewing human ES cell-derived neural stem cell with potential for in vitro instruction and synaptic integration. Proc Natl Acad Sci U S A. 106: 3225-30.

4. Koch P, Tamboli IY, Mertens J, Wunderlich P, Ladewig J, Stuber K, et al. (2012) Presenilin-1 L166P mutant human pluripotent stem cell-derived neurons exhibit partial loss of gamma-secretase activity in endogenous amyloid-beta generation. Am J Pathol. 180: 2404-16.

5. Wood SJ, Maleeff B, Hart T, Wetzel R (1996) Physical, morphological and functional differences between ph 5.8 and 7.4 aggregates of the Alzheimer's amyloid peptide Abeta. J Mol Biol. 256: 870-7.
